# Performance, egg quality and organ traits of laying hens fed black soldier fly larvae products

**DOI:** 10.1016/j.psj.2024.104229

**Published:** 2024-08-19

**Authors:** Anna Dörper, Gerrit Gort, Jan van Harn, Dennis G.A.B. Oonincx, Marcel Dicke, Teun Veldkamp

**Affiliations:** ⁎ Laboratory of Entomology, Wageningen University & Research, 6700AA Wageningen, The Netherlands; † Biometris, Wageningen University & Research, 6700AA Wageningen, The Netherlands; ‡ Wageningen Livestock Research, Wageningen University & Research, 6700AH Wageningen, The Netherlands; § Animal Nutrition Group, Wageningen University & Research, 6708 WD Wageningen, The Netherlands

**Keywords:** laying hen performance, egg quality, organ size, insect as feed, *Hermetia illucens*

## Abstract

Due to consumer demands and institutional pressure, the egg production sector, is looking for alternative protein sources for laying hen feed to support more sustainable, circular production. black soldier fly (**BSF**) larvae could be used as a protein source. In addition to protein the larvae contain large quantities of fat and can either be fed to laying hens unprocessed (alive) or processed (meal and oil). The current study was performed with 560 Brown Nick laying hens from 20 to 27 wk of age. The laying hens were divided over 5 treatments, each replicated 8 times. Treatments consisted of standard laying hen feed (control) and standard feed in which soybean meal was partly exchanged with live BSF larvae or BSF larvae meal and oil combined, at 2 inclusion levels. During the experiment production parameters, egg-quality, and length and weight of various organs were measured.

Laying hens fed BSF larvae products consumed less feed compared to those of the control group. Most egg production parameters were similar, however laying hens fed diets with BSF larvae meal plus oil produced eggs with lower egg weight during the last 2 wk of the experiment, compared to the control group. All egg-quality characteristics remained the same across treatments, except for darker yolk colors when feeding BSF meal and oil and high inclusion of live BSF larvae. This is a favorable characteristic for European consumers. The weight of intestinal organs was largely unaffected by the treatments. The jejunum and ileum weight of laying hens fed live larvae was lower compared to the control group. As FCRs were similar or improved compared to the control group, we assume that nutrient utilization was not impaired. For most detected differences the type of BSF larvae product (live larvae or meal plus oil) rather than inclusion level was of significance.

## INTRODUCTION

In livestock feed, soybean meal is one of the most used protein sources. The European Union accounts for 13% (2020–2022) of the global soy demand and is highly dependent on external sources from Brazil and Argentina (Kuepper and Stravens, 2022; OECD/FAO, 2023). Based on the origin, the import of soybean meal comes with long distance transportation and is often connected to deforestation of the amazon. Also, societal concerns arise increasingly from the production of soybeans due to the common use of genetically modified seeds. Particularly in the European Union, retailer certification programs for animal products derived from animals fed on non-GMO diets are driving a shift to other protein sources in feed (OECD/FAO, 2023). Also elsewhere for example China, scenarios are developed for the use of nonsoybean protein sources (Bai et al., 2023). In the EU, approximately 53% of the soybean meal used in livestock feed is used in poultry feed. In 2020, in the EU and UK 12.560,000 tons soybean meal were used in broiler feed and 3,532,000 tons soybean meal were used in laying hen feed (Kuepper and Stravens, 2022), highlighting the need to find alternative protein sources, especially in these sectors.

In Europe, common alternative local sources are rapeseed meal and sunflower seed meal (FEFAC, 2024), both suitable and used as vegetal protein sources in laying hen feed (OECD/FAO, 2023). Both sources contain less true protein (sum of measured amino acids) (395 g/kg DM and 377 g/kg DM, respectively) compared to soybean meal (526 g/kg DM) and show on average an amino acid digestibility coefficient of 79 and 83, respectively. The values are a bit lower compared to soybean meal having an average amino acid digestibility coefficient of 86 (CVB, 2022). Insects are common nutrient sources for poultry in nature (Collias and Saichuae, 1967). Alternatively, black soldier fly (**BSF**) larvae could also be used as protein sources in poultry feed. For instance, the red jungle fowl, the ancestor of domestic laying hens, consumes a wide range of insects from ants to cockroaches (Collias and Saichuae, 1967). Larvae of the BSF are not yet commonly used in feed, so to our knowledge there are no tables of standard nutritional values as are available for common feed ingredients. However, whole BSF larvae and larvae meal have been reported to contain 360 to 678 g/kg DM true protein (Dörper et al., 2024; Finke, 2013; Janssen et al., 2017) and to have an average amino acid digestibility coefficient ranging from 77 to 85 (Mahmoud et al., 2023; Schiavone et al., 2017). Aside from protein, BSF larvae also contain considerable amounts of fat, rich in lauric acid (17–45 % of total fatty acids) (Dörper et al., 2024; Kim et al., 2020; Li et al., 2022). Lauric acid, but also antimicrobial peptides, and chitin in BSF larvae have been suggested to have health promoting properties for livestock (Dörper et al., 2021; Gasco et al., 2018). Based on their nutritional profiles, unprocessed live BSF larvae or processed products such as BSF larvae meal and oil could fulfill different functional roles in laying hens.

The use of BSF larvae products, such as live larvae and processed larvae (meal and oil) included in laying hen diets at levels up to 17% as fed, can affect performance and egg quality differently in laying hens. The inclusion of live BSF larvae in layer diets did not result in altered layer performance and egg quality compared to the control group (Star et al., 2020). The use of BSF larvae meal in laying hen diets has in some cases resulted in similar performance such as feed intake, body weight, feed conversion ratio (Bovera et al., 2018; Zawisza et al., 2023), and laying rate (Liu et al., 2021; Zawisza et al., 2023), or egg quality such as egg weight (Bovera et al., 2018; Secci et al., 2018; Zawisza et al., 2023) compared to laying hens fed a control diet. Whereas others reported improved performance such as lower feed intake (Marono et al., 2017; Patterson et al., 2021; Wamai et al., 2024), lower feed conversion ratios (Bovera et al., 2018; Marono et al., 2017), and higher laying rates (Bovera et al., 2018; Wamai et al., 2024) and improved egg quality such as higher egg mass (Bovera et al., 2018), egg weight (Liu et al., 2021; Secci et al., 2020; Wamai et al., 2024), and darker egg yolks (Patterson et al., 2021; Secci et al., 2018) compared to laying hens of the control group. The use of BSF larvae oil as a replacement for soybean oil in laying hen diets did not alter performance such as the feed intake, body weight, and feed conversion ratio (Kim et al., 2022; Patterson et al., 2021), and did not affect egg quality such as egg mass and egg weight (Kim et al., 2022; Patterson et al., 2021), while yolk color was darker (Kim et al., 2022; Patterson et al., 2021) compared to laying hens of the control group. In some cases, the effect on laying hen performance and egg quality was dependent on the inclusion level of the BSF larvae product in the diet (Bovera et al., 2018; Patterson et al., 2021; Secci et al., 2020; Wamai et al., 2024). As performance of laying hens and digestion of feed are closely related, effects on performance might be explained by the development of internal organs. This should therefore be investigated.

Based on the inconsistent results, it seems difficult to get a clear idea of the potential of BSF larvae products in laying hen diets on performance and egg quality. In the cited literature single products were tested in experiments. To compare the potential of processed and unprocessed BSF larvae products it is essential to evaluate their effects on laying hen performance in 1 study. Therefore, we aimed here to investigate the effect of using live or processed (meal and oil) BSF larvae in laying hen diets on performance and egg quality, using 2 inclusion levels. The aim was to replace 5% or 10% of laying hens dry matter feed intake with live black soldier fly larvae. The BSF larvae meal and oil was mixed to mirror the crude protein and crude fat content provided with the replacement of 5% and 10% dry matter feed intake with live BSF larvae.

## MATERIALS AND METHODS

### Ethical Approval

The project approval was granted by the Central Authority for Scientific Procedures on Animals (CCD) and the Animal welfare body of Wageningen University evaluated the experimental procedures with the application number AVD40100202010104. The experiment was then conducted from September until December 2022.

### Housing

For the trial, 560 18.5-wk-old Brown Nick (H&N International, Cuxhaven, Germany) laying hens were obtained from Agromix Broederij en Opfokintegratie B.V. (Lunteren, the Netherlands) and housed in the research facility “Carus” of Wageningen University (Wageningen, the Netherlands). The laying hens were divided over 5 treatment groups, each with 8 replicates containing 14 laying hens, leading to a total of 40 pens (each 2 m^2^; more details below). The pens were evenly divided over 4 rooms, leading to 10 pens per room. In each room, 5 consecutive pens were considered 1 block. In each block every treatment group was represented once. The order of the treatment groups was randomized per block. At arrival, the laying hens were weighed and distributed across pens to achieve uniform weight distribution (average laying hen weight per pen ± 3 %). After arrival, a 12-d-adaptation period followed which was divided into 2 phases. The first phase, which lasted 6 d, was dedicated to adapting to the new environment. The second phase, which also lasted 6 d, was dedicated to adapting to the experimental diets. The laying hens were 20 wk old when the experiment started. The experimental period lasted 7 wk. At the start of the experimental period the light program was set to 13 h light, which was increased weekly by 1 h until 16 h was reached at wk 4 of the experimental period. The temperature was kept between 21 and 22°C.

The pens were 2 meters long, 1 meter wide, and 2 meters high (7 laying hens/m^2^) and could be entered through a door on the front side. All pens were equipped with 2 perches (1 m long), a drinker line (1 m long, 5 nipple drinkers with drip cups), a pecking stone, a nest box (93 cm long x 38 cm deep x 58 cm high) placed outside the pen, but accessible from the pen, a hanging feed trough (⌀ 38 cm) for mash feed and a feed trough for live larvae (1.3 m long x 4.25 cm deep x 2.13 cm high). Both feeders were hanging and the laying hens could move below them. However, subtracting the feeder space from the floor space results in a space allowance of 7.6 laying hens per m^2^. Wood shavings (1.6 kg/m^2^) were used as bedding material.

### Diet Formulation

For accurate feed formulation the chemical composition of main feed ingredients (corn and wheat), main protein sources (soybean meal, sunflower seed meal, rapeseed meal, wheat middling's, BSF larvae meal), and main fat sources (soybean oil, BSF larvae oil) were analyzed by Agrolab Lufa GmbH (Kiel, Germany) accredited according to DIN EN ISO/IEC 17025:2018 (Supplementary Tables S1–3). Analyses were performed according to standard procedures (ISO 2009, DIN EN 15621:2017-10, VDLUFA III, 10.5.1:1976). The feed was formulated (Table 1) together with the provider, Research Diet Services B.V. (Wijk bij Duurstede, The Netherlands). The 5 diets were formulated isoenergetic and based on equal levels of digestible essential amino acids (Table 2). The chemical composition of live BSF larvae was thereby considered. The trial was conducted with 5 treatments. The control group received a standard corn-wheat-based laying hen diet. The second treatment group (L-low) received as replacement of 5% of the dry matter feed intake live BSF larvae and a complementing mash feed, the third treatment group (MO-low) received a mash diet with BSF larvae meal and oil in the diet to mimic the nutritional value of the live BSF larvae provided in the second treatment. The fourth treatment group (L-high) received as replacement of 10% of the DM feed intake live BSF larvae and a complementing mash feed. The fifth treatment group (MO-high) received a mash diet with BSF larvae meal and oil in the diet to mimic the nutritional value of the live BSF larvae provided in the fourth treatment. After diet production a sample of all diets was nutritionally analyzed by Agrolab Lufa GmbH accredited according to DIN EN ISO/IEC 17025:2018 (Kiel, Germany) (Supplementary Table S 4–6). Analyses were performed according to standard procedures (ISO 2009, DIN EN 15621:2017-10, VDLUFA III, 10.5.1:1976).

**Table 1:** Ingredients (g/kg as fed, if not indicated otherwise) of all experimental rations.

Ingredients	Control^1^	L-low^2^	MO-low^3^	L-high^4^	MO-high^5^
Corn	462.17	472.20	469.60	482.27	477.02
Wheat	150.00	150.00	150.00	150.00	150.00
Soybean meal	111.50	60.50	60.75	9.50	10.00
Sunflower seed meal	100.00	100.00	100.00	100.00	100.00
Wheat middling	30.00	30.00	30.00	30.00	30.00
Rapeseed meal	10.00	10.00	10.00	10.00	10.00
Soybean oil	26.00	16.00	14.00	6.00	2.00
Premix (corn)	5.00	5.00	5.00	5.00	5.00
Diamol			5.00		10.00
Limestone 1.2-2 mm	80.00	80.00	80.00	80.00	80.00
Limestone fine (38% Ca)	9.70	9.15	9.55	8.60	9.40
Monocalcium phosphate	5.00	4.70	4.65	4.40	4.30
Sodium hydrogen carbonate	3.80	3.90	3.90	4.00	4.00
Sodium chloride (Salt)	1.00	0.85	0.80	0.70	0.60
Potassium carbonate		0.50	0.55	1.00	1.10
L-lysine HCl	2.10	2.33	2.38	2.55	2.65
DL-methionine	2.05	2.18	2.20	2.30	2.35
L-threonine	0.80	0.93	0.95	1.05	1.10
L-isoleucine (90%)	0.35	0.53	0.60	0.70	0.85
L-arginine	0.50	1.18	1.18	1.85	1.85
L-tryptophan		0.03	0.03	0.05	0.05
Phytase enzyme	0.03	0.03	0.03	0.03	0.03
Live BSF larvae (DM basis)^6^		50.00		100.00	
BSF larvae meal			40.50		81.00
BSF larvae oil			8.35		16.70

BSF = black soldier fly.

1 Control = Standard corn-wheat-based layer diet.

2 L-low = 5% of the daily dry matter feed intake is replaced by live BSF larvae.

3 MO-low = BSF larvae meal and oil in the diet mimic the nutritional value of the live larvae in treatment L-low.

4 L-high = 10% of the daily dry matter feed intake is replaced by live BSF larvae.

5 MO-high = BSF larvae meal and oil in the diet mimic the nutritional value of the live larvae in treatment L-high.

6 The live BSF larvae were considered during the diet formulation as a product with 100% dry matter. During the trial, the moisture content of the live larvae taken into account. We calculated how much fresh matter live larvae is needed to satisfy 5 and 10 % of laying hens dry matter feed intake.

**Table 2:** Calculated composition (g/kg as fed) of the experimental rations and diets.

Calculated contents	Control^1^	L-low^2^	MO-low^3^	L-high^4^	MO-high^5^
Dry matter	891.3	895.3	893.0	899.2	894.7
Ash	122.7	121.6	126.5	120.5	130.4
Crude protein	155.4	155.4	155.5	155.5	155.5
Digestible indispensable AA					
Lysine	6.9	6.9	6.9	6.9	6.9
Methionine	4.2	4.3	4.3	4.4	4.5
Cysteine	2.0	1.8	1.8	1.7	1.7
Methionine and Cysteine	6.1	6.1	6.1	6.1	6.1
Threonine	4.8	4.8	4.8	4.8	4.8
Tryptophan	1.6	1.5	1.5	1.5	1.5
Isoleucine	5.5	5.5	5.5	5.5	5.5
Arginine	8.3	8.3	8.3	8.3	8.3
Valine	6.0	6.2	6.2	6.4	6.4
Crude fat	55.9	58.0	55.9	60.1	56.0
Crude fiber	40.0	42.2	42.2	44.4	44.4
Carbohydrates	514.7	516.3	511.1	518.0	507.4
Starch	399.3	405.7	404.0	412.2	408.8
Sugars	28.8	24.4	24.4	19.9	19.9
Neutral detergent fiber	125.4	122.2	122.0	119.1	118.6
Acid detergent fiber	52.7	50.5	50.4	48.3	48.1
Calcium	37.0	37.0	37.0	37.0	37.0
Phosphorus	4.9	5.0	5.0	5.0	5.0
Potassium	6.6	6.3	6.3	6.0	6.0
Phosphorus available	3.3	3.5	3.5	3.7	3.8
Magnesium	1.7	1.5	1.5	1.4	1.4
Chloride	2.0	2.0	2.0	2.0	2.0
Sodium	1.5	1.5	1.5	1.5	1.5
Metabolizable energy (MJ/kg)	11.9	11.9	11.9	11.9	11.9

AA = Amino acids.

1 Control = Standard corn-wheat-based layer diet.

2 L-low = 5% of the daily dry matter feed intake is replaced by live BSF larvae.

3 MO-low = BSF larvae meal and oil in the diet mimic the nutritional value of the live larvae in treatment L-low.

4 L-high = 10% of the daily dry matter feed intake is replaced by live BSF larvae.

5 MO-high = BSF larvae meal and oil in the diet mimic the nutritional value of the live larvae in treatment L-high.

### Larvae Products

Larvae products (live BSF larvae, BSF larvae meal, and BSF larvae oil) were supplied by Protix BV (Dongen, the Netherlands). BSF larvae meal and oil were incorporated in the mash diet, while live BSF larvae were fed via an automatic larvae dispenser (Dörper et al., 2023) into a tube system that led into a feed trough feeder (Dörper et al., 2024). The dispensers were filled every morning from 7:30-8:00h. The last larvae were released between 16:00h and 18:00h. Live BSF larvae were received once per week in crates (60*40*16 cm) with sawdust. Upon arrival, the crates were inspected for any visible substrate residues, which were removed if present. An additional 200 g of sawdust was added to each crate to secure a dry environment during storage in a climate chamber (10°C and 70%). Based on the expected dry matter feed intake of the laying hens and their feed intake in the previous week (H&N International GmbH, 2022), the quantities of live BSF larvae per pen were prepared 1 d in advance. The approximate required amount of larvae (considering 67.6 % moisture (Dörper et al., 2024)) was taken from the climate chamber, sieved (4×4 mm mesh) to remove the sawdust, weighed into portions per pen, then again stored in the climate chamber until the next morning. A subsample of larvae was collected each day during larval preparation and stored at -20°C for nutritional analysis of a pooled sample at the end of the study (Supplementary Tables S1–3).

### Measurements

#### Performance and Egg Production

The laying hens were individually weighed at arrival and at the end of the experiment. Based on the data the average laying hen weight and body weight variation (standard deviation) per pen were calculated. During the experiment the mash provided and mash leftover was weighed per pen on a weekly basis. The intake of mash and live larvae were added to calculate the weekly dry matter feed intake (**DMFI**). Moreover, the date of the first egg and the number of eggs per day per pen were recorded. The hens’ daily egg production (**HDEP**) per week was calculated by based on number of eggs laid per pen and number of laying hen per pen. Three times a week the individual egg weight of all first grade eggs (excluding second grade eggs, such as broken, cracked, dirty, and double yolk eggs) of that day was measured to determine the average egg weight and egg mass, per pen, per week. The egg weight represents the average weight per egg. Egg mass was calculated by multiplying the weekly HDEP and average egg weight. The feed conversion ratio (**FCR**) was calculated per dozen eggs (FCR_dozen_) and per egg mass unit (FCR_mass_). Laying hens started to lay irregularly at 21 wk of age. Egg production stabilized at 23 wk of age. Therefore, data of 23 wk of age and onwards were used for FCR analyses.

#### Egg Composition and Quality

During wk 5 of the experimental period (25-wk-old laying hens), 8 eggs per pen were collected and shipped to the University of Turin (Grugliasco, Italy) for proximate analyses of yolk and albumen. In wk 7 (27-wk-old laying hens), ten eggs were collected per pen and shipped to the Institute for Egg Quality-management (Amersfoort, the Netherlands). Here they were stored below 10°C for maximally 5 d before the Haugh unit was assessed. Egg weight, index (the ratio between egg weight and length, multiplied by 100), eggshell weight, breaking strength (FUTURA Egg-Shell-Tester, Bröring Technology GmbH, Lohne, Germany), thickness (eggshell thickness gauge, Bröring Technology GmbH, Lohne, Germany), color (shell color reflectometer, TSS, York, United Kingdom), albumen weight, albumen height (albumen height gauge, TSS, York, United Kingdom), Haugh unit, yolk weight, yolk height (albumen height gauge, TSS, York, United Kingdom), and yolk color (DSM-Firmenich YolkFan, DSM, Heerlen, the Netherlands) were determined.

#### Dissection

During the individual weighing of the laying hens at the end of the experimental period 3 animals close to the average pen weight ±3% were color marked. Two laying hens selected for dissection were marked with a purple spray and 1 laying hen that functioned as a reserve animal, was marked with a yellow spray. Laying hens of the first twenty pens were dissected on 1 d and the others the day after. Prior to dissection the laying hens were euthanized by intravenous pentobarbital injection. The weights of the crop, gizzard, proventriculus, liver, spleen, and the weight and length of the duodenum (from pyloric region to end of the pancreatic loop), jejunum (from the end of the pancreatic loop to Meckel's diverticulum), ileum (from Meckel's diverticulum to ileo-caecal junction), and caeca (from ileo-caecal junction to the tip of the caeca) were measured. Digesta and adherent fat were removed before weighing the organs (Amerah et al., 2008; Perera et al., 2021).

#### Statistics

All data were analyzed with the open-source software R (R version 4.1.2, R Core Team, 2021). DMFI, egg weight, HDEP, egg mass and FCRs are repeated measurement per pen over time.

The DMFI a linear mixed effect model (**LMM**) (using R library, lme4: Bates et al., 2015), wherease for the egg weight, HDEP, egg mass, and FCR generalized linear mixed models (**GLMM**) (using R library glmmTMB: Brooks et al., 2017) were used. The model accounted for varying dispersion across different weeks and utilized the normal distribution with a logit link function. Treatment (Control, L-low, MO-low, L-high, and MO-high), wk (1–7), and their interaction were added in the models having fixed effects. Pen number (1–40) was added having random effects, to account for the weekly repeated measurements, and room nested in week was added having random effects.

Average body weight per laying hen per pen, body weight variation per pen, age when first egg laid per pen, and data from the proximate analyses of eggs are single measurements obtained at the pen level and were analyzed with an LMM (using R library, lme4: Bates et al., 2015). The model contained treatment having fixed effects and room having random effects. At the end of the trial, the body weight of 1 laying hen was noted to be 5.3 kg. As this was approximately 3 times the average weight, it was almost certainly incorrect and therefore excluded from the analyses.

Egg quality characteristics are measurements on multiple eggs per pen and were analyzed with the LMM (using R library, lme4: Bates et al., 2015). Relative organ weight and length are measurements on multiple hens per pen, and were was analyzed with a GLMM (using R library glmmTMB: Brooks et al., 2017) with the beta distribution and logit link. Treatment was modeled having fixed effects and room and pen number having random effects. Data of yolk color was reciprocally transformed to satisfy the model assumptions.

Additional linear hypotheses were tested with the models employing customized contrasts for the effect of diet type (control, L, and MO), inclusion level (low and high), and their interaction. For models containing week effects, customized contrasts for the interactions: diet type and week, inclusion and week, and diet type, inclusion and week were also created. At 2 instances a laying hen escaped their home pen and was found in the adjacent pen. Therefore, the data of pen 32 and 33 in wk 5 and 6, and data of pen 36 and 37 in wk 6 and 7 were removed from the analyses. The fit of all models was evaluated with residual diagnostic plots (DHARMa: Hartig, 2022). Significance was declared at *P* < 0.05. Multiple comparisons were performed, in case of significance, with adjusted probability values using the multivariate t distribution. The results presented in tables and figures show descriptive statistics of the raw data: ordinary means and standard errors.

## RESULTS

The average body weight per laying hen per pen (Table 3) was significantly affected by diet type, inclusion level and laying hen age. At arrival, at 18.5 wk of age, laying hens across all treatment groups had by design almost equal body weights across all treatment groups. At the end of the experimental period, at 27 wk of age, laying hens fed low BSF larvae meal and oil and high live BSF larvae levels and in their diet were significantly heavier than laying hens fed high inclusion of BSF larvae meal and oil. However, there were no differences detected between laying hens fed with diets or rations containing BSF larvae products compared to laying hens of the control group. Variation in body weight was not significantly different across laying hens of different treatments, diet types and inclusion levels (Table 3).

**Table 3:** Body weight (g/laying hen) and body weight variation (standard deviation) of Brown Nick laying hens at 18.5 and 27 wk of age.

		Body weight (g/laying hen)		Body weight variation (standard deviation)^6^	
Laying hen age (wk)		18.5	27	18.5	27
Control		1252 ± 5.1	1765 ± 8.2^ab^	89 ± 5.1	120 ± 9.4
L-low		1248 ± 5.1	1757 ± 12.8^ab^	83 ± 4.3	118 ± 11.8
MO-low		1257 ± 6.3	1771 ± 8.3^a^	84 ± 6.6	111 ± 11.2
L-high		1251 ± 6.5	1776 ± 12.8^a^	76 ± 7.1	127 ± 11.2
MO-high		1249 ± 7.0	1732 ± 8.6^b^	87 ± 3.0	124 ± 6.3
	DF^5^	*P*-values			
Treatment^1^	4	0.850	0.022	0.557	0.831
Diet type^2^	2	0.856	0.249	0.557	0.877
Inclusion^3^	1	0.640	0.277	0.345	0.288
Diet type*Inclusion^4^	1	0.373	0.005	0.778	0.860

1 Treatments (Control = Standard corn-wheat based layer diet, L-low = 5% of the daily dry matter feed intake is replaced by live black soldier fly (BSF) larvae, MO-low = BSF larvae meal and oil in the diet mimic the nutritional value of the live larvae in treatment L-low, L-high = 10% of the daily dry matter feed intake is replaced by live BSF larvae, MO-high = BSF larvae meal and oil in the diet mimic the nutritional value of the live larvae in treatment L-high). Every treatment had 8 replicate pens.

2 Diet types (Control, L= diets with live larvae, MO= diets with BSF larvae meal and oil).

3 Inclusion (low= diets with low inclusion of BSF larvae products, and high =diets with high inclusion of BSF larvae products).

4 The control group was excluded in the contrasts of the interactions between diet type and inclusion level.

5 DF = degrees of freedom.

6 Body weight variation = body weight variation per pen expressed as the standard deviation. Data is presented as ordinary mean ± standard error. Different lower-case letters in a row indicate significant differences (*P* < 0.05).

**Figure 1:**
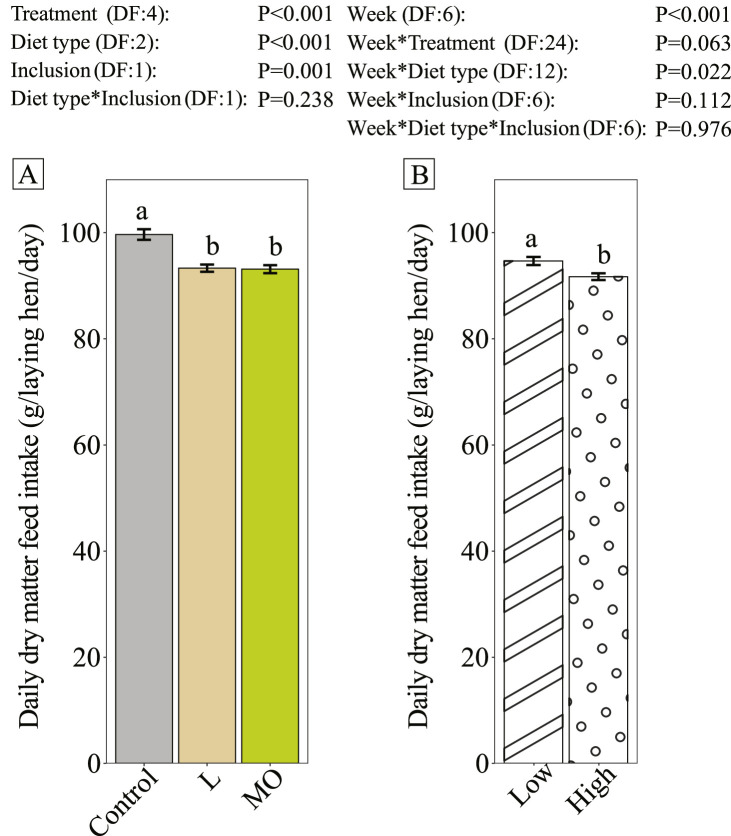
Daily dry matter feed intake (g/laying hen) per wk of Brown Nick laying hens during the experiment period (age 21 to 27 wk). Data is presented as ordinary mean ± standard error. Different lower-case letters per graph indicate significance (*P* < 0.05). The bars in panel A indicate the different diet types (Control, L= diets with live larvae, MO= diets with black soldier fly (BSF) larvae meal and oil). The bars in panel B indicate different inclusion levels (low = low inclusion of BSF larvae products, high= high inclusion of BSF larvae products). Every treatment had 8 replicate pens. The control group was excluded in the contrasts of the interaction between diet type and inclusion level.

Laying hens fed diets or rations with BSF larvae products consumed significantly less feed (6.5%) than the control group (Figure 1). Moreover, a higher inclusion level further decreased DMFI significantly, but the interaction between diet type and inclusion was not significant.

**Figure 2:**
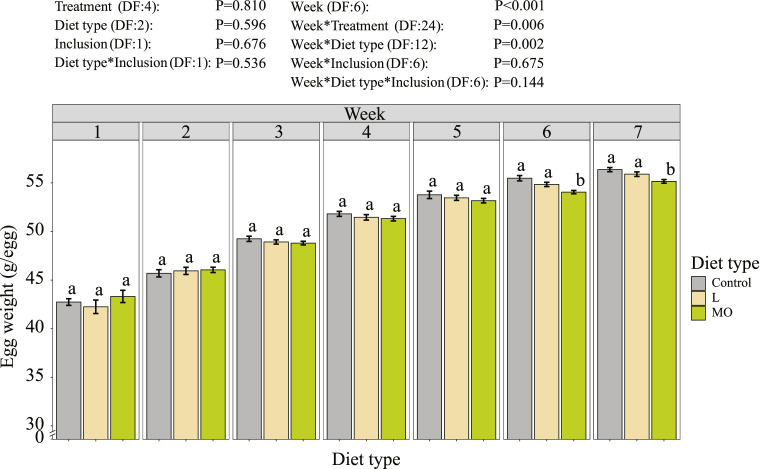
Egg weight (g/egg) per week of Brown Nick laying hens between age of 21 to 27 wk. Data is presented as ordinary mean ± standard error. Different lower-case letters per graph indicate significance (*P* < 0.05). The bars indicate the different diet types (Control, L= diets with live larvae, MO = diets with BSF larvae meal and oil). The control group had 8 replicate pens, the other diet types had 16 replicate pens. The control group was excluded in the contrasts of the interaction between diet type and inclusion level.

Furthermore, laying hens fed live BSF larvae laid their first egg about 2 d earlier than laying hens fed the control diet, while laying hens fed BSF larvae meal and oil were in between (Table 4). The parameters egg mass and HDEP were not affected by treatment, diet type or inclusion level. Egg weight was significantly affected by the diet type, dependent on the experimental week (Figure 2). During wk 1 to 5, the egg weights of laying hens across all diet types were similar. During wk 6 and 7 laying hens fed BSF larvae meal and oil in their diet produced eggs with significantly lower egg weight than those of the control group and those fed live BSF larvae.

**Table 4:** Egg production parameters of Brown Nick laying hens at 21 and 27 wk of age.

		First laid egg (d/laying hen)^1^	Egg mass (g)	Hen day egg production %
Control		146.5 ± 0.7^b^	43.1 ± 2.1	74.5 ± 4.7
L		144.8 ± 0.5^a^	40.6 ± 1.7	74.4 ± 3.3
MO		145.3 ± 0.4^ab^	38.9 ± 1.7	73.1 ± 3.3
	DF^6^	*P*-values		
Treatment^2^	4	0.125	0.371	0.691
Diet type^3^	2	**0.044**	0.530	0.939
Inclusion^4^	1	0.349	0.091	0.154
Diet type*Inclusion^5^	1	0.814	0.716	0.799
Week	6		**<0.001**	**<0.001**
Week*Treatment	24		0.347	0.724
Week*Diet type	12		0.565	0.875
Week*Inclusion	6		0.224	0.171
Week*Diet type*Inclusion	6		0.276	0.724

1 First laid egg since the laying hens hatched.

2 Treatments (Control = Standard corn-wheat based layer diet, L-low = 5% of the daily dry matter feed intake is replaced by live black soldier fly (BSF) larvae, MO-low = BSF larvae meal and oil in the diet mimic the nutritional value of the live larvae in treatment L-low, L-high = 10% of the daily dry matter feed intake is replaced by live BSF larvae, MO-high = BSF larvae meal and oil in the diet mimic the nutritional value of the live larvae in treatment L-high). Every treatment had 8 replicate pens.

3 Diet types (Control, L= diets with live larvae, MO= diets with BSF larvae meal and oil).

4 Inclusion (low= diets with low inclusion of BSF larvae products, and high =diets with high inclusion of BSF larvae products).

5 The control group was excluded in the contrasts of the interactions between diet type and inclusion level.

6 DF = degrees of freedom. Data is presented as ordinary mean ± standard error. Different lower-case letters in a row indicate significant differences (*P* < 0.05). P-values < 0.05 are highlighted in bold.

**Figure 3:**
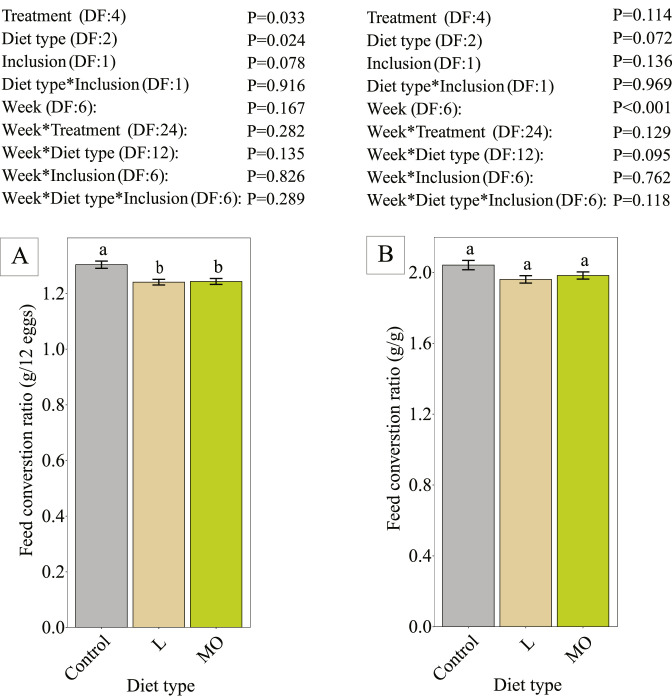
Feed conversion ratio calculated per dozen eggs produced (panel A) and based on egg mass (panel B) of Brown Nick laying hens during the experiment period (age 23 to 27 wk). Data is presented as ordinary mean ± standard error. Different lower-case letters per graph indicate significance (*P* < 0.05). The bars indicate the different diet types (Control, L= diets with live larvae, MO = diets with BSF larvae meal and oil). Every treatment had 8 replicate pens. The control group was excluded in the contrasts of the interaction between diet type and inclusion level.

The FCR_dozen_ was significantly influenced by diet type (Figure 3). Laying hens fed BSF larvae products had a 4.7% lower FCR compared to the control. The FCR_mass_ was not significantly different between any of the treatments, diet types, and inclusion levels. The FCRs might be further influenced if also the first 2 wk of production are included in FCR analysis.

Eggs were collected from 25-wk-old hens to determine the proximate composition of yolk and albumen. These parameters were not significantly different across treatments, diet types and inclusion levels, except for albumen dry matter content (Table 5). The albumen dry matter content in eggs of the control group laying hens was significantly lower than eggs from laying hens fed BSF meal and oil in their diet. Egg albumen dry matter content was intermediate for hens provided with live BSF larvae. At 27 wk of age, eggs were collected to quantify egg quality characteristics (Table 6). Yolk color of eggs from laying hens fed low or high inclusion of BSF meal and oil, and from laying hens fed high inclusion of BSF live larvae, was significantly darker than those from the control group. Furthermore, the effect of diet type significantly depended on inclusion level; a high inclusion of live BSF larvae in the diet led to darker yolks than low inclusion of live BSF larvae. Other egg quality characteristics were not significantly affected.

**Table 5:** Egg yolk and albumen proximate composition (g dry matter, if not indicated otherwise) of 25-wk-old Brown Nick laying hens.

		Yolk				Albumen	
		Dry matter (g)	Ash	Crude protein	Crude fat	Dry matter (g)	Crude protein
Control		46.9 ± 0.24	3.5 ± 0.07	33.9 ± 0.16	58.3 ± 0.11	11.8 ± 0.13^a^	85.9 ± 0.13
L		47.0 ± 0.17	3.6 ± 0.06	34.0 ± 0.07	58.1 ± 0.08	12.1 ± 0.08^ab^	86.1 ± 0.08
MO		47.0 ± 0.16	3.6 ± 0.07	34.0 ± 0.11	58.0 ± 0.07	12.3 ± 0.09^b^	86.1 ± 0.09
	DF^5^	*P*-values					
Treatment^1^	4	0.989	0.966	0.778	0.202	**0.027**	0.654
Diet type^2^	2	0.922	0.765	0.621	0.102	**0.005**	0.654
Inclusion^3^	1	0.715	0.914	0.861	0.818	0.829	0.327
Diet type* Inclusion^4^	1	0.978	0.912	0.388	0.248	0.641	0.864

1 Treatments (Control = Standard corn-wheat based layer diet, L-low = 5% of the daily dry matter feed intake is replaced by live black soldier fly (BSF) larvae, MO-low = BSF larvae meal and oil in the diet mimic the nutritional value of the live larvae in treatment L-low, L-high = 10% of the daily dry matter feed intake is replaced by live BSF larvae, MO-high = BSF larvae meal and oil in the diet mimic the nutritional value of the live larvae in treatment L-high). Every treatment had 8 replicate pens.

2 Diet types (Control, L = diets with live larvae, MO= diets with BSF larvae meal and oil).

3 Inclusion (low= diets with low inclusion of BSF larvae products, and high =diets with high inclusion of BSF larvae products).

4 The control group was excluded in the contrasts of the interactions between diet type and inclusion level.

5 DF = degrees of freedom. Data is presented as ordinary mean ± standard error. Different lower-case letters in a column indicate significant differences (*P* < 0.05). P-values < 0.05 are highlighted in bold.

**Table 6:** Egg quality characteristics of 27-wk-old Brown Nick laying hens.

		Weight (g/egg)	Index^6^	Shell color	Shell weight (g/egg)	Shell thickness (mm)	Shell breaking strength (N)	Albumen weight (g)	Haugh unit	Yolk color	Yolk weight (g/egg)	Yolk height (mm)
Control		56.5 ± 0.48	78.2 ± 0.27	19.4 ± 0.35	6.6 ± 0.07	0.38 ± 0.002	58.2 ± 0.89	31.7 ± 0.30	83.9 ± 0.67	3.0 ± 0.05^c^	17.5 ± 0.16	19.8 ± 0.15
L-low		56.8 ± 0.47	78.1 ± 0.28	19.3 ± 0.39	6.6 ± 0.06	0.39 ± 0.002	57.4 ± 0.92	32.0 ± 0.30	83.0 ± 0.63	3.3 ± 0.08^bc^	17.6 ± 0.16	19.9 ± 0.15
MO-low		55.7 ± 0.44	77.5 ± 0.26	19.3 ± 0.39	6.5 ± 0.07	0.38 ± 0.003	56.9 ± 1.04	31.2 ± 0.28	83.4 ± 0.64	3.8 ± 0.13^ab^	17.2 ± 0.14	19.8 ± 0.16
L-high		56.4 ± 0.41	78.3 ± 0.27	18.7 ± 0.40	6.6 ± 0.06	0.38 ± 0.002	57.3 ± 0.92	31.6 ± 0.26	84.4 ± 0.48	3.8 ± 0.11^a^	17.5 ± 0.14	19.7 ± 0.14
MO-high		56.6 ± 0.39	78.3 ± 0.23	19.2 ± 0.32	6.6 ± 0.05	0.38 ± 0.003	57.7 ± 0.79	31.8 ± 0.25	85.3 ± 0.51	3.6 ± 0.11^ab^	17.4 ± 0.15	19.7 ± 0.14
	DF^5^	*P*-values										
Treatment^1^	4	0.489	0.267	0.813	0.657	0.451	0.900	0.536	0.608	**<0.001**	0.583	0.950
Diet type^2^	2	0.573	0.444	0.735	0.619	0.262	0.716	0.710	0.834	**0.001**	0.583	0.931
Inclusion^3^	1	0.579	0.100	0.453	0.473	0.406	0.696	0.809	0.137	0.112	0.374	0.485
Diet type*Inclusion^4^	1	0.161	0.338	0.549	0.338	0.604	0.642	0.129	0.847	**0.011**	0.935	0.820

1 Treatments (Control = Standard corn-wheat based layer diet, L-low = 5% of the daily dry matter feed intake is replaced by live black soldier fly (BSF) larvae, MO-low = BSF larvae meal and oil in the diet mimic the nutritional value of the live larvae in treatment L-low, L-high = 10% of the daily dry matter feed intake is replaced by live BSF larvae, MO-high = BSF larvae meal and oil in the diet mimic the nutritional value of the live larvae in treatment L-high). Every treatment had 16 replicates.

2 Diet types (Control, L= diets with live larvae, MO= diets with BSF larvae meal and oil).

3 Inclusion (low= diets with low inclusion of BSF larvae products, and high = diets with high inclusion of BSF larvae products).

4 The control group was excluded in the contrasts of the interactions between diet type and inclusion level.

5 DF = degrees of freedom.

6 Index = The egg index is calculated by dividing the egg width by the egg length multiplied by 100. Data is presented as ordinary mean ± standard error. Different lower-case letters in a column indicate significant differences (*P* < 0.05). P-values < 0.05 are highlighted in bold.

After 7 wk of trial, laying hens were dissected to measure the length and weight of intestinal organs (Table 7, Table 8). Gizzard and proventriculus weight were significantly lower at high inclusion levels than at low levels of the BSF products, but similar to the weight of the control group. Feeding live BSF larvae to laying hens led to significantly lower jejunum and ileum weights than the control group. The inclusion of BSF meal and oil in laying hen diets also led to significantly lower jejunum weight compared to the control group. The relative length of intestinal organs were similar over diet types except for a significantly shorter ileum in laying hens fed live BSF larvae, compared to laying hens of the control group.

**Table 7:** Relative intestinal organ weight (% live weight, if not indicated otherwise) of 28-wk-old Brown Nick laying hens.

		Live weight (g)	Crop	Gizzard	Proventriculus	Duodenum	Jejunum	Ileum	Caecum	Liver	Spleen
Control		1796.8 ± 11.4	0.32 ± 0.02	1.42 ± 0.04	0.34 ± 0.01	0.45 ± 0.06	1.00 ± 0.03^b^	0.55 ± 0.02^b^	0.21 ± 0.01	2.47 ± 0.06	0.10 ± 0.01
Live		1788.0 ± 9.6	0.30 ± 0.01	1.46 ± 0.04	0.34 ± 0.01	0.42 ± 0.04	0.89 ± 0.02^a^	0.49 ± 0.01^a^	0.20 ± 0.01	2.36 ± 0.05	0.10 ± 0.00
MO		1776.0 ± 7.7	0.31 ± 0.01	1.42 ± 0.03	0.35 ± 0.01	0.38 ± 0.04	0.88 ± 0.02^a^	0.52 ± 0.01^ab^	0.20 ± 0.01	2.46 ± 0.05	0.10 ± 0.00
Low		1790.9 ± 8.3	0.31 ± 0.01	1.49 ± 0.04^b^	0.35 ± 0.00^b^	0.38 ± 0.04	0.90 ± 0.02	0.51 ± 0.01	0.20 ± 0.01	2.43 ± 0.06	0.10 ± 0.00
High		1773.1 ± 8.9	0.30 ± 0.01	1.39 ± 0.03^a^	0.34 ± 0.01^a^	0.42 ± 0.04	0.87 ± 0.02	0.50 ± 0.01	0.21 ± 0.01	2.39 ± 0.05	0.10 ± 0.00
	DF^5^	*P*-values									
Treatment^1^	4	0.058	0.507	0.071	0.054	0.217	**0.007**	**0.021**	0.848	0.340	0.340
Diet type^2^	2	0.170	0.224	0.662	0.083	0.292	**0.002**	**0.006**	0.600	0.194	0.319
Inclusion^3^	1	0.061	0.621	**0.044**	**0.039**	0.261	0.182	0.381	0.557	0.507	0.154
Diet type*Inclusion^4^	1	0.129	0.806	0.057	0.688	0.136	0.622	0.395	0.964	0.354	0.713

1 Treatments (Control = Standard corn-wheat based layer diet, L-low = 5% of the daily dry matter feed intake is replaced by live black soldier fly (BSF) larvae, MO-low = BSF larvae meal and oil in the diet mimic the nutritional value of the live larvae in treatment L-low, L-high = 10% of the daily dry matter feed intake is replaced by live BSF larvae, MO-high = BSF larvae meal and oil in the diet mimic the nutritional value of the live larvae in treatment L-high). Every treatment had 16 replicates.

2 Diet types (Control, L= diets with live larvae, MO = diets with BSF larvae meal and oil).

3 Inclusion (low= diets with low inclusion of BSF larvae products, and high =diets with high inclusion of BSF larvae products).

4 The control group was excluded in the contrasts of the interactions between diet type and inclusion level.

5 DF = degrees of freedom. Data is presented as ordinary mean ± standard error. Different lower-case letters in a column indicate significant differences (*P* < 0.05). P-values < 0.05 are highlighted in bold.

**Table 8:** Relative intestinal organ length (% live weight) of 28-wk-old Brown Nick laying hens.

		Duodenum	Jejunum	Ileum	Caecum
Control		1.79 ± 0.04	3.92 ± 0.09	3.83 ± 0.08^b^	0.87 ± 0.03
L		1.72 ± 0.02	3.80 ± 0.06	3.61 ± 0.05^a^	0.81 ± 0.02
MO		1.72 ± 0.02	3.84 ± 0.06	3.68 ± 0.04^ab^	0.86 ± 0.02
	DF^5^	*P*-values			
Treatment^1^	4	0.449	0.815	0.128	0.123
Diet type^2^	2	0.177	0.594	**0.046**	0.050
Inclusion^3^	1	0.840	0.805	0.578	0.616
Diet type*Inclusion^4^	1	0.702	0.503	0.399	0.288

1 Treatments (Control = Standard corn-wheat based layer diet, L-low = 5% of the daily dry matter feed intake is replaced by live black soldier fly (BSF) larvae, MO-low = BSF larvae meal and oil in the diet mimic the nutritional value of the live larvae in treatment L-low, L-high = 10% of the daily dry matter feed intake is replaced by live BSF larvae, MO-high = BSF larvae meal and oil in the diet mimic the nutritional value of the live larvae in treatment L-high). Every treatment had 16 replicates.

2 Diet types (Control, L= diets with live larvae, MO= diets with BSF larvae meal and oil).

3 Inclusion (low= diets with low inclusion of BSF larvae products, and high =diets with high inclusion of BSF larvae products).

4 The control group was excluded in the contrasts of the interactions between diet type and inclusion level.

5 DF = degrees of freedom. Data is presented as ordinary mean ± standard error. Different lower-case letters in a column indicate significant differences (*P* < 0.05). P-values < 0.05 are highlighted in bold.

**Figure 4:**
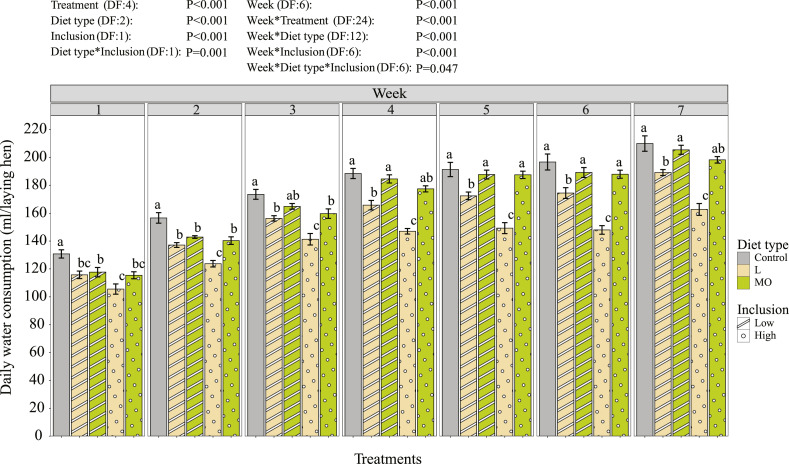
Daily water consumption (mL/laying hen) of 23 to 27-wk-old Brown Nick laying hens. Data is presented as ordinary mean ± standard error. Different lower-case letters per graph indicate significant differences (*P* < 0.05). The bars indicate the different treatments (Control, L-low = 5% of the daily dry matter feed intake is replaced by live black soldier fly (BSF) larvae, MO-low = BSF larvae meal and oil in the diet mimic the nutritional value of the live larvae in treatment L-low, L-high = 10% of the daily dry matter feed intake is replaced by live BSF larvae, MO-high = BSF larvae meal and oil in the diet mimic the nutritional value of the live larvae in treatment L-high). Every treatment had 8 replicate pens. The control group was excluded in the contrasts of the interaction between diet type and inclusion level.

The water consumption of the laying hens significantly differed over treatments and diet types, depending on the inclusion level and wk (Figure 4). Laying hens fed live BSF larvae consumed significantly less water compared to the control group in all weeks. From the second wk onwards, laying hens fed on a diet with high inclusion of live BSF larvae consumed significantly less water those provided with a low inclusion of BSF live larvae. However, water consumption of laying hens fed low or high inclusion of BSF meal and oil was similar

## DISCUSSION

In the current study we compared the use of live and processed BSF larvae products at 2 inclusion levels in laying hen diets to evaluate their effects on laying hen performance, egg quality, and organ weight and length.

During the 7-wk trial, with a replacement of 5% or 10% of the DMFI, live larvae were always completely consumed. This indicates the laying hen's eagerness to eat live larvae and their continued attractiveness throughout the study. The total DMFI (mash feed and larvae) was lower for laying hens fed BSF larvae products compared to the control group. Also in other studies, laying hens reduced their feed intake of concentrate feed when they were provided with live BSF larvae (Star et al., 2020; Tahamtani et al., 2021). However, in those studies the reduction in consumption of mash feed and live larvae combined was not statistically analyzed.(Star et al., 2020; Tahamtani et al., 2021). In the study of Star et al. (2020) live larvae replaced 3% of the daily DMFI. Based on the information provided on the DM of larvae and concentrate feed, the combined consumption of mash feed and live larvae was numerically lower for laying hens fed live BSF larvae (113 g/laying hen/d) compared to the control group (118 g/laying hen/d), indicating similar results as in our study. In studies using up to 4.5% as fed BSF larvae oil (Kim et al., 2022; Patterson et al., 2021) or up to 16 % as fed BSF larvae meal (Bovera et al., 2018; Patterson et al., 2021) in laying hen diets, feed intake was similar to the control group. To our knowledge, there is no study available using meal and oil together in laying hen diets, considering isocaloric and isonitrogenous diets or balanced digestible amino acid profiles during feed formulation. The finding of our study could indicate that the combination of both products is necessary to achieve positive effect on performance. In the current study, results showed significant differences between diet types for FCR_dozen_ and a tendency for different FCR_mass_ between laying hens fed different diet types. Most likely, the result is based on the lower feed intake of laying hens fed with live BSF larvae and BSF meal and oil, while remaining similar production compared to laying hens of the control group. In other studies, the FCR of laying hens fed live BSF larvae was either not determined or was based on the consumption of mash feed, excluding the live BSF larvae (Star et al., 2020; Tahamtani et al., 2021).

As expected, feeding live larvae to laying hens decreased water consumption. From 2 wk of the experiment onwards, the high inclusion of live larvae also led to a lower water consumption from nipple drinkers than low larvae inclusion. The live BSF larvae used in the trial contained high quantities of moisture (69.4 %). Laying hens seemed to be able to adjust their water consumption according to the inclusion of live larvae. Also in other studies feeding insect meal and insect oil as components of slow-growing broiler or laying hen diets led to reduced water consumption (Dörper et al., 2024; Heuel et al., 2022; Nieto et al., 2023, 2022). Previous studies have demonstrated a strong interaction between water consumption and feed intake in chickens (Bierer et al., 1966). Low feed intake leads to low water consumption (Bierer et al., 1966), as laying hens fed BSF larvae products in their diet consumed less feed this could lead to an effect on water consumption.

The body weight and variation in body weight was similar between laying hens of the control group and other treatment groups and diet types. It was assumed that competition and different hierarchy status between laying hens could lead to uneven intake of live larvae between individuals. The resulting imbalanced nutrient intake would lead to a greater variation in body weight. Hence, the data suggests that all laying hens were able to consume an equal portion of the live larvae. In our experimental setup, the space per laying hen to feed (in terms of length at the feed trough) is the sum of the length at the round feeder for mash feed and the length of the feed trough for live larvae. Together, the feed troughs provide 17.8 cm per laying hen. According to the EU Directive (1999/74/EC), laying hens must be provided with at least 10 cm at a linear feeder and at least 4 cm at a circular feeder per laying hen. Laying hens had therefore under the current experimental conditions more space at the feed troughs compared to conventional settings. It would be interesting to investigate how space at the feed troughs, but also different shapes of the feed troughs, influence the effect of feeding live larvae on the behavior of laying hens.

Egg production parameters such as HDEP, egg mass, and egg quality characteristics remained similar between laying hens of the control group and other treatments and diet types. This is in line with other studies investigating the effect of live BSF larvae (Star et al., 2020), BSF larvae meal (Patterson et al., 2021), and BSF oil (Kim et al., 2022; Patterson et al., 2021) on layer performance and egg quality characteristics. However, laying hens that consumed diets including BSF larvae meal and oil produced eggs with lower egg weight during the final 2 wk of the experiment. The reason for the reduced egg weight could be the lower feed intake of the laying hens and therefore lower intake of methionine (Cadirci 2012). Similar results were found when laying hens were fed full-fat BSF larvae meal (Marono et al., 2017), yet also the FCR of those hens was lower which is environmentally and economically favorable. In our study, the FCR_dozen_ was significantly reduced and the FCR_mass_ tended to be lower for laying hens fed with BSF larvae products compared to the control group.

Yolk color was affected by the treatment. High inclusion of live larvae and inclusion of low or high levels of meal and oil resulted in darker yolk colors compared to the control group. In most European countries, consumers prefer dark egg yolk colors (Hernández, 2005). Therefore natural or artificial pigments are commonly added to the feed (Hernández, 2005; Marounek and Pebriansyah, 2018). The use of BSF larvae products could reduce the need for such additions.

Laying hens fed live larvae showed a lower relative jejunum and ileum weight and a lower relative ileum length. Increased fiber content such as cellulose and wood shavings in broiler diets can reduce the length of the small intestine (Amerah et al., 2009). A shorter small intestine might be caused by a lower nutrient density of the feed, reducing the surface area required for absorption (Amerah et al., 2009). The achieved results may be related to the content of chitin in the diets. Chitin is embedded in a matrix with other nutrients like proteins, lipids and minerals (Józefiak et al., 2016), which could hinder absorption of these nutrients in the small intestine. Nonetheless, as the FCR_dozen_ and FCR_mass_ indicate improved and similar conversion of nutrients compared to the control group, chitin digestion and absorption of nutrients might have taken place at the cecal level due to microbial activity.

To conclude, BSF larvae products in layer diets reduced feed intake compared to laying hens of the control group. While most egg production parameters remained unchanged, laying hens provided with BSF larvae meal and oil produced eggs with a lower weight during the last 2 wk of the experimental period, compared to the control group. This could potentially have a negative impact on production, and therefore it would be of interest to investigate if the decreasing influence on egg weight will persist over the full production cycle of the laying hens. The weight of intestinal organs was largely unaffected by the treatments. The jejunum and ileum weight of laying hens fed live larvae was lower compared to the control group. However, because the FCR_dozen_ and FCR_mass_ of the laying hens was improved or similar when larvae products were fed compared to the control group, it is assumed that nutrient utilization was not impaired. It would be of great interest to investigate how the current findings translate into practice. For production parameters such as FCR and egg production, it seems more relevant to focus on the different BSF larvae products rather than inclusion levels.

## DISCLOSURES

The authors declare the following financial interests/personal relationships which may be considered as potential competing interests: Marcel Dicke reports financial support was provided by Dutch Research Council. The project is funded by the Dutch Research Council (NWO; NWA programme, InsectFeed project, NWA.1160.18.144). If there are other authors, they declare that they have no known competing financial interests or personal relationships that could have appeared to influence the work reported in this paper.

## Appendix Supplementary materials

Image, application 1

## Footnotes

Supplementary material associated with this article can be found in the online version at doi:10.1016/j.psj.2024.104229.
