# Supplementary material for: Performance, egg quality and organ traits of laying hens fed black soldier fly larvae products
Source: Poult Sci. 2024 Aug 19;103(11):104229. doi: 10.1016/j.psj.2024.104229 (PMC11414572; doi:10.1016/j.psj.2024.104229)
Supplement: Supplementary file 1 [file mmc1.docx]

**Supplementary information**

**Table S 1:** Analyzed proximate and mineral composition (% as fed) of ration ingredients

|  | Corn | Wheat | Wheat Middings | Soybean meal | Rapeseed meal | Sunflower seed meal | BSF larvae meal | Frozen BSF larvae | BSF larvae oil | Soybean oil |
| --- | --- | --- | --- | --- | --- | --- | --- | --- | --- | --- |
| Proximate composition |  |  |  |  |  |  |  |  |  |  |
| Moisture | 12.8 | 12.5 | 11.3 | 11.5 | 10.6 | 11.2 | 6.3 | 69.4 |  |  |
| Crude ash | 1.4 | 1.7 | 5.0 | 7.1 | 6.9 | 7.3 | 6.8 | 1.9 |  |  |
| Crude protein (Nx6,25) | 7.5 | 12.0 | 14.8 | 47.8 | 33.5 | 36.3 | 53.7 | 13.7 |  |  |
| Crude fat | 4.1 | 2.0 | 3.9 | 3.5 | 4.9 | 2.3 | 12.7 | 9.8 | >99.5 | >99.5 |
| Crude fibre | 2.3 | 2.5 | 7.7 | 3.7 | 11.8 | 17.9 | 9.6 | 2.3 |  |  |
| Calculated values |  |  |  |  |  |  |  |  |  |  |
| N-free substances | 71.9 | 69.3 | 57.3 | 26.4 | 32.3 | 25.0 | 10.9 | 2.9 |  |  |
| Minerals |  |  |  |  |  |  |  |  |  |  |
| Sodium | <0.01 | <0.01 | 0.03 | 0.02 | 0.12 | 0.02 | 0.16 | 0.04 | <0.01 | <0.01 |
| Potassium | 0.35 | 0.37 | 1.25 | 2.24 | 1.23 | 1.40 | 1.30 | 0.38 | <0.01 | <0.01 |
| Magnesium |  |  |  |  |  |  |  | 0.10 |  |  |
| Calcium | 0.02 | 0.07 | 0.14 | 0.36 | 0.79 | 0.63 | 0.73 | 0.32 | <0.01 | <0.01 |
| Phosphorus | 0.23 | 0.30 | 0.96 | 0.65 | 1.02 | 1.18 | 1.01 | 0.27 | <0.01 | <0.01 |
| Chloride | 0.12 | 0.12 | 0.12 | 0.10 | <0.10 | 0.12 | 0.31 | 0.11 | <0.10 | <0.10 |
| If a table cell is empty, it means that the corresponding value was not measured. The symbol "<". in the result column means that the substance concerned was not quantifiable as it was below the limit of detection. | | | | | | | | | | |

**Table S 2:** Analyzed amino acid composition (% as fed) of ration ingredients

|  | Corn | Wheat | Wheat Middings | Soybean meal | Rapeseed meal | Sunflower seed meal | BSF larvae meal | Frozen BSF larvae |
| --- | --- | --- | --- | --- | --- | --- | --- | --- |
| Lysine | 0.23 | 0.32 | 0.56 | 2.75 | 1.75 | 1.15 | 3.07 | 0.69 |
| Methionine | 0.13 | 0.16 | 0.19 | 0.60 | 0.60 | 0.70 | 0.80 | 0.17 |
| Cysteine | 0.15 | 0.26 | 0.30 | 0.62 | 0.73 | 0.51 | 0.48 | 0.09 |
| Asparaginic acid | 0.49 | 0.55 | 0.98 | 5.33 | 2.26 | 2.89 | 4.60 | 0.97 |
| Threonine | 0.25 | 0.30 | 0.43 | 1.75 | 1.35 | 1.15 | 1.94 | 0.43 |
| Serine | 0.30 | 0.48 | 0.55 | 2.19 | 1.25 | 1.24 | 1.98 | 0.45 |
| Glutamic acid | 1.26 | 3.22 | 2.71 | 8.65 | 5.29 | 6.50 | 5.71 | 1.20 |
| Proline | 0.58 | 1.01 | 0.83 | 2.33 | 1.96 | 1.34 | 3.08 | 0.71 |
| Glycine | 0.28 | 0.47 | 0.73 | 1.99 | 1.63 | 1.96 | 2.73 | 0.68 |
| Alanine | 0.51 | 0.41 | 0.66 | 2.03 | 1.38 | 1.39 | 3.58 | 0.92 |
| Valine | 0.34 | 0.50 | 0.69 | 2.35 | 1.68 | 1.69 | 3.20 | 0.74 |
| Isoleucine | 0.23 | 0.39 | 0.46 | 2.31 | 1.32 | 1.40 | 2.27 | 0.59 |
| Leucine | 0.78 | 0.72 | 0.86 | 3.61 | 2.18 | 2.04 | 3.45 | 0.87 |
| Tyrosine | 0.27 | 0.29 | 0.33 | 1.51 | 0.85 | 0.66 | 2.84 | 0.61 |
| Phenylalanine | 0.31 | 0.49 | 0.56 | 2.38 | 1.22 | 1.44 | 2.04 | 0.49 |
| Histidine | 0.19 | 0.25 | 0.37 | 1.20 | 0.83 | 0.82 | 1.48 | 0.30 |
| Arginine | 0.31 | 0.51 | 0.91 | 3.17 | 1.83 | 2.52 | 2.35 | 0.46 |
| Tryptophan | 0.07 | 0.15 | 0.26 | 0.60 | 0.43 | 0.47 | 0.73 | 0.18 |
| Sum amino acids | 6.68 | 10.50 | 12.40 | 45.40 | 28.50 | 29.90 | 46.30 | 10.60 |
|  | | | | | | | | |

**Table S 3:** Analyzed fatty acid composition (% as fed) of ration ingredients

|  | Corn | Wheat | Wheat middlings | Soybean meal | Rapeseed meal | Sunflower seed meal | BSF larvae meal | Frozen BSF larvae | BSF larvae oil | Soybean oil |
| --- | --- | --- | --- | --- | --- | --- | --- | --- | --- | --- |
| Capric acid | <0.1 | <0.1 | <0.1 | <0.1 | <0.1 | <0.1 | 0.8 | 0.9 | 1.0 | <0.1 |
| Lauric acid | <0.1 | <0.1 | 1.4 | <0.1 | 0.2 | 0.5 | 35.3 | 39.7 | 43.5 | <0.1 |
| Myristic acid | <0.1 | 0.1 | 0.6 | <0.1 | 0.2 | 0.4 | 8.4 | 9.3 | 9.9 | <0.1 |
| Myristoleic acid | <0.1 | <0.1 | <0.1 | 0.1 | <0.1 | 0.2 | 0.2 | 0.2 | 0.3 | <0.1 |
| Pentadecanoic acid | <0.1 | 0.1 | 0.1 | <0.1 | 0.1 | <0.1 | 0.1 | 0.1 | 0.1 | <0.1 |
| Palmitic acid | 12.1 | 17.1 | 17.1 | 14.7 | 8.2 | 10.8 | 16.3 | 15.9 | 14.7 | 9.8 |
| Palmitoleinic acid | 0.1 | 0.2 | 0.2 | 0.1 | 1.0 | 0.3 | 3.1 | 2.7 | 3.0 | <0.1 |
| Hexadecatrienic acid | <0.1 | <0.1 | <0.1 | <0.1 | 0.1 | <0.1 | <0.1 | <0.1 | <0.1 | <0.1 |
| Margaric acid | <0.1 | 0.1 | 0.2 | 0.1 | 0.1 | <0.1 | 0.2 | 0.2 | 0.1 | 0.1 |
| Stearic acid | 1.9 | 1.2 | 1.0 | 3.7 | 1.7 | 4.1 | 3.4 | 2.8 | 2.4 | 4.0 |
| Octadecenoic acid | <0.1 | <0.1 | <0.1 | <0.1 | 0.3 | 0.4 | <0.1 | <0.1 | <0.1 | <0.1 |
| Oleic acid | 27.1 | 14.4 | 14.3 | 19.8 | 45.5 | 45.1 | 13.5 | 10.7 | 9.2 | 18.1 |
| Cis-vaccenic acid | 0.7 | 1.0 | 1.0 | 1.7 | 9.1 | 0.9 | 0.3 | 0.4 | 0.3 | 1.1 |
| Octadecadienoic acid | <0.1 | <0.1 | <0.1 | 0.1 | 0.3 | <0.1 | <0.1 | <0.1 | <0.1 | <0.1 |
| Linoleic acid | 55.1 | 59.1 | 56.7 | 51.5 | 25.0 | 34.2 | 16.5 | 15.4 | 14.2 | 56.5 |
| Octadecatetrienic acid | <0.1 | <0.1 | 0.1 | 0.3 | 0.6 | <0.1 | <0.1 | <0.1 | <0.1 | <0.1 |
| Alpha-linolenic acid | 1.5 | 4.4 | 5.2 | 6.3 | 5.8 | 0.5 | 1.4 | 1.3 | 1.2 | 8.9 |
| Arachic acid | 0.5 | 0.2 | 0.2 | <0.1 | <0.1 | 0.4 | <0.1 | <0.1 | 0.1 | 0.4 |
| Eicosenoic acid | 0.4 | 0.7 | 0.8 | 0.5 | 0.7 | 0.7 | 0.1 | <0.1 | 0.1 | 0.2 |
| Eicosadienic acid | <0.1 | 0.1 | 0.1 | <0.1 | <0.1 | <0.1 | <0.1 | <0.1 | <0.1 | <0.1 |
| Behenic acid | 0.2 | 0.3 | 0.3 | 0.5 | 0.3 | 0.8 | <0.1 | <0.1 | <0.1 | 0.4 |
| Lignoceric acid | 0.3 | 0.3 | 0.3 | 0.2 | 0.2 | 0.4 | <0.1 | <0.1 | <0.1 | 0.1 |
| Nervonic acid | <0.1 | <0.1 | <0.1 | <0.1 | 0.1 | <0.1 | <0.1 | <0.1 | <0.1 | <0.1 |
| The symbol "<". in the result column means that the substance concerned was not quantifiable as it was below the limit of detection. | | | | | | | | | | |

**Table S 4**: Analyzed proximate and mineral composition (% as fed) of mash feed for 19- to 27-week-old Brown Nick laying hens

|  | Control^1^ | L-low^2^ | MO-low^3^ | L-high^4^ | MO-high^5^ |
| --- | --- | --- | --- | --- | --- |
| Proximate composition |  |  |  |  |  |
| Moisture | 10.6 | 11.0 | 10.7 | 10.9 | 10.7 |
| Crude ash | 13.2 | 12.5 | 13.1 | 14.3 | 12.3 |
| Crude protein (Nx6,25) | 15.5 | 14.0 | 15.4 | 12.3 | 15.1 |
| Crude fat, total | 5.5 | 4.3 | 5.3 | 3.3 | 5.1 |
| Crude fibre | 4.5 | 4.4 | 4.6 | 4.1 | 4.5 |
| Calculated values |  |  |  |  |  |
| N-free substances | 50.7 | 53.8 | 50.9 | 55.1 | 52.3 |
| Minerals |  |  |  |  |  |
| Sodium | 0.20 | 0.16 | 0.13 | 0.20 | 0.13 |
| Potassium | 0.66 | 0.60 | 0.67 | 0.54 | 0.62 |
| Calcium | 4.48 | 3.97 | 3.66 | 4.99 | 3.55 |
| Phosphorus | 0.51 | 0.48 | 0.51 | 0.47 | 0.51 |
| Chloride | 0.16 | 0.15 | 0.17 | 0.19 | 0.17 |
| ^1^ Control = Standard corn-wheat-based layer diet  ^2^ L-low = 5% of the daily dry matter feed intake is replaced by live BSF larvae  ^3^ MO-low = BSF larvae meal and oil in the diet mimic the nutritional value of the live larvae in treatment L-low  ^4^ L-high = 10% of the daily dry matter feed intake is replaced by live BSF larvae  ^5^ MO-high = BSF larvae meal and oil in the diet mimic the nutritional value of the live larvae in treatment L-high | | | | | |

**Table S 5:** Analyzed amino acid composition (% as fed) of mash feed for 19- to 27-week-old Brown Nick laying hens

|  | Control^1^ | L-low^2^ | MO-low^3^ | L-high^4^ | MO-high^5^ |
| --- | --- | --- | --- | --- | --- |
| Lysine | 0.83 | 0.71 | 0.80 | 0.66 | 0.78 |
| Methionine | 0.49 | 0.47 | 0.45 | 0.50 | 0.44 |
| Cysteine | 0.25 | 0.23 | 0.25 | 0.22 | 0.23 |
| Asparaginic acid | 1.27 | 1.05 | 1.26 | 0.87 | 1.12 |
| Threonine | 0.61 | 0.55 | 0.61 | 0.51 | 0.60 |
| Serine | 0.64 | 0.56 | 0.65 | 0.48 | 0.60 |
| Glutamic acid | 2.81 | 2.54 | 2.85 | 2.30 | 2.48 |
| Proline | 0.86 | 0.81 | 1.00 | 0.75 | 0.91 |
| Glycine | 0.66 | 0.59 | 0.72 | 0.55 | 0.69 |
| Alanine | 0.69 | 0.62 | 0.80 | 0.58 | 0.80 |
| Valine | 0.69 | 0.61 | 0.76 | 0.54 | 0.73 |
| Isoleucine | 0.61 | 0.56 | 0.65 | 0.50 | 0.64 |
| Leucine | 1.12 | 1.02 | 1.19 | 0.91 | 1.13 |
| Tyrosine | 0.44 | 0.40 | 0.49 | 0.34 | 0.54 |
| Phenylalanine | 0.69 | 0.61 | 0.69 | 0.53 | 0.63 |
| Histidine | 0.36 | 0.32 | 0.40 | 0.29 | 0.37 |
| Arginine | 0.96 | 0.91 | 1.03 | 0.88 | 0.93 |
| Tryptophan | 0.17 | 0.14 | 0.17 | 0.13 | 0.16 |
| ^1^ Control = Standard corn-wheat-based layer diet  ^2^ L-low = 5% of the daily dry matter feed intake is replaced by live BSF larvae  ^3^ MO-low = BSF larvae meal and oil in the diet mimic the nutritional value of the live larvae in treatment L-low  ^4^ L-high = 10% of the daily dry matter feed intake is replaced by live BSF larvae  ^5^ MO-high = BSF larvae meal and oil in the diet mimic the nutritional value of the live larvae in treatment L-high | | | | | |

**Table S 6**: Analyzed fatty acid composition (% as fed) of mash feed for 19 to 27 week-old Brown Nick laying hens

|  | Control^1^ | L-low^2^ | MO-low^3^ | L-high^4^ | MO-high^5^ |
| --- | --- | --- | --- | --- | --- |
| Capric acid | <0.1 | <0.1 | 0.2 | <0.1 | 0.5 |
| Lauric acid | <0.1 | <0.1 | 9.9 | <0.1 | 20.2 |
| Myristic acid | 0.1 | <0.1 | 2.4 | <0.1 | 4.7 |
| Myristoleic acid | <0.1 | <0.1 | <0.1 | <0.1 | 0.1 |
| Pentadecanoic acid | <0.1 | <0.1 | <0.1 | <0.1 | 0.1 |
| Palmitic acid | 11.5 | 11.9 | 12.8 | 12.4 | 14.1 |
| Palmitoleinic acid | <0.1 | 0.1 | 0.8 | 0.1 | 1.7 |
| Margaric acid | 0.1 | <0.1 | 0.1 | <0.1 | 0.1 |
| Stearic acid | 3.2 | 2.9 | 2.8 | 2.5 | 2.5 |
| Oleic acid | 22.3 | 23.2 | 20.5 | 25.3 | 18.5 |
| cis-vaccenic acid | 1.2 | 1.1 | 0.9 | 1.0 | 0.6 |
| Octadecadienoic acid | <0.1 | <0.1 | <0.1 | 0.1 | <0.1 |
| Linoleic acid | 54.3 | 54.3 | 44.4 | 53.1 | 33.8 |
| alpha-linolenic acid | 5.7 | 4.8 | 3.7 | 3.5 | 1.9 |
| Arachic acid | 0.4 | 0.4 | 0.3 | 0.4 | 0.3 |
| Eicosenoic acid | 0.3 | 0.3 | 0.3 | 0.4 | 0.3 |
| Behenic acid | 0.4 | 0.3 | 0.2 | 0.3 | 0.2 |
| Lignoceric acid | 0.2 | 0.2 | 0.2 | 0.3 | 0.2 |
| The symbol "<". in the result column means that the substance concerned was not quantifiable as it was below the limit of detection.  ^1^ Control = Standard corn-wheat-based layer diet  ^2^ L-low = 5% of the daily dry matter feed intake is replaced by live BSF larvae  ^3^ MO-low = BSF larvae meal and oil in the diet mimic the nutritional value of the live larvae in treatment L-low  ^4^ L-high = 10% of the daily dry matter feed intake is replaced by live BSF larvae  ^5^ MO-high = BSF larvae meal and oil in the diet mimic the nutritional value of the live larvae in treatment L-high | | | | | |
